# Epigenetic Silencing of MicroRNA-126 Promotes Cell Growth in Marek’s Disease

**DOI:** 10.3390/microorganisms9061339

**Published:** 2021-06-21

**Authors:** Isabelle Gennart, Astrid Petit, Laetitia Wiggers, Srđan Pejaković, Nicolas Dauchot, Sylvie Laurent, Damien Coupeau, Benoît Muylkens

**Affiliations:** 1 Integrated Veterinary Research Unit (URVI), Namur Research Institute for Life Sciences (NARILIS), Université de Namur, 61 Rue de Bruxelles, 5000 Namur, Belgium; igennart@hotmail.com (I.G.); laetitia.wiggers@unamur.be (L.W.); spejakovic1@gmail.com (S.P.); damien.coupeau@unamur.be (D.C.); 2 Unit of Research in Plant Cellular and Molecular Biology (URBV), Université de Namur, 61 Rue de Bruxelles, 5000 Namur, Belgium; dauchot.nicolas@gmail.com; 3 Département Santé Animale, Institut National de la Recherche pour l’Agriculture, l’Alimentation et l’Environnement (INRAE), Centre Val de Loire, 37380 Nouzilly, France; sylvie.laurent@inrae.fr

**Keywords:** Gallid alphaherpesvirus 2, Marek’s disease, herpesvirus, epigenetic silencing, micro-RNA-126, EGFL-7, tumorigenesis, CRK

## Abstract

During latency, herpesvirus infection results in the establishment of a dormant state in which a restricted set of viral genes are expressed. Together with alterations of the viral genome, several host genes undergo epigenetic silencing during latency. These epigenetic dysregulations of cellular genes might be involved in the development of cancer. In this context, Gallid alphaherpesvirus 2 (GaHV-2), causing Marek’s disease (MD) in susceptible chicken, was shown to impair the expression of several cellular microRNAs (miRNAs). We decided to focus on gga-miR-126, a host miRNA considered a tumor suppressor through signaling pathways controlling cell proliferation. Our objectives were to analyze the cause and the impact of miR-126 silencing during GaHV-2 infection. This cellular miRNA was found to be repressed at crucial steps of the viral infection. In order to determine whether miR-126 low expression level was associated with specific epigenetic signatures, DNA methylation patterns were established in the miR-126 gene promoter. Repression was associated with hypermethylation at a CpG island located in the miR-126 host gene epidermal growth factor like-7 (*EGFL-7*). A strategy was developed to conditionally overexpress miR-126 and control miRNAs in transformed CD4+ T cells propagated from Marek’s disease (MD) lymphoma. This functional assay showed that miR-126 restoration specifically diminishes cell proliferation. We identified CT10 regulator of kinase (CRK), an adaptor protein dysregulated in several human malignancies, as a candidate target gene. Indeed, CRK protein levels were markedly reduced by the miR-126 restoration.

## 1. Introduction

MicroRNAs (miRNAs) are a class of endogenous, small non-coding RNAs of about 22 nucleotides (nt) in length that govern post-transcriptional repression of target genes by binding to the 3′ untranslated region (UTR) or gene bodies [1]. Generally, miRNAs negatively regulate gene expression by decreasing messenger RNA (mRNA) stability and interfering with translation [2]. They play important roles in several biological processes such as cell proliferation, development, differentiation, and tumorigenesis [3,4]. An increasing number of studies show aberrantly expressed miRNAs in cancers. Some miRNAs act as oncogenes [5,6], while others show tumor suppressor activity [7]. Misexpression of miRNAs mediates neoplastic transformation [8] and is intimately linked to lymphoma development in hematologic malignancies [9,10,11]. MiRNAs have been reported to be involved in virus-induced, in addition to non-infectious, forms of cancers [12]. As several human viruses are associated with cancer development, animal models are needed to explore mechanisms and processes linking viral infection and oncogenesis. 

In this context, Marek’s disease (MD) appeared as a unique natural model for herpesvirus-induced lymphomagenesis [13]. MD is a highly contagious and lymphoproliferative neoplastic disease, causing CD4+ T lymphoma in chicken. This disease was first reported by a Hungarian veterinarian, Joszef Marek, over a century ago. Since the late 1960s, vaccines were used to control the disease, but there is strong evidence that vaccination has contributed to the evolution of more virulent strains of GaHV-2 [14]. Nowadays, MD is still considered worldwide as a severe threat to avian health and poultry production. The disease is induced by the infection of susceptible birds with oncogenic strains of Gallid alphaherpesvirus 2 (GaHV-2), also known as Marek’s disease virus serotype 1 (MDV1). This herpesvirus shares several properties with human herpesviruses (such as the Epstein Barr virus (HHV-4) and Kaposi’s sarcoma-associated herpes virus (HHV-8)) associated with cancer development in humans under specific conditions in latently infected cells. The pathology induced by GaHV-2 begins in an early stage with transient immunosuppression followed by lymphoma formation in visceral organs of susceptible chickens [15]. The viral life cycle contains four stages: (1) the early productive phase 2 to 7 days post-infection (dpi); (2) the latent phase from 7 dpi until death; (3) the late productive phase (from 14 dpi); and (4) the tumorigenesis phase (from 21 dpi).

During tumorigenesis, GaHV-2 regulates viral and host gene expression through different viral encoded proteins, such as the Meq-oncogene, and a large set of viral non-coding RNAs. Among them, viral miRNAs participate in the transformation of latently infected cells [16]. The importance of cellular miRNAs in host–pathogen interactions has also been identified in the last decade [17,18]. Among the host miRNAs dysregulated by GaHV-2, miR-26a was shown to be repressed and plays a role as a tumor suppressor, while miR-221/222 and miR-21 were observed to be overexpressed during the viral oncogenesis, enhancing cell proliferation [5,7] and interfering with apoptosis [6]. In this context, miR-126 is under investigation as this non-coding RNA is repressed in several human cancers [19].

MiR-126 is a highly conserved gene, and it has an increased expression in vascularized tissues [20]. Knock-out studies in zebrafish and mice suggested a significant role of miR-126 in angiogenesis and vascular integrity [21,22]. This miRNA is an intronic miRNA localized in the host gene termed epidermal growth factor like-7 (*EGFL-7*), playing a role in vasculogenesis [20]. Previous studies showed that miR-126 expression inhibits tumor cell proliferation, invasion, and the epithelial–mesenchymal transition (EMT) process by targeting oncogenic genes such as v-crk avian sarcoma virus CT10 oncogene homolog (*CRK*) [23,24,25]. In numerous cancers, miR-126 was shown to be downregulated, such as in cancers of endocrine glands and genital tracts, gastrointestinal cancers, and cancers of the respiratory system, suggesting its tumor suppressor function [19].

Aberrant miR-126 expression in several human cancers was shown to be related to epigenetic modifications. Hypermethylation was associated with the repression of miR-126 in various cancers such as prostate and bladder cancers, colorectal carcinoma, glioma, and lung cancer [26,27,28,29]. This observation is in agreement with previous observations showing that almost all types of cancers harbor hundreds of genes with abnormal gain in DNA methylation [30]. Moreover, numerous intragenic tumor suppressor miRNAs are known to be regulated by epigenetic mechanisms and are usually silenced by excessive DNA methylation, resulting in tumorigenesis [31].

This study tackles the question of miR-126 repression in the course of GaHV-2 induced lymphomagenesis. MiR-126 expression level was determined at key steps of the viral infection and was correlated with the DNA methylation pattern on two CpG islands of its host gene. To address the impact of miR-126 silencing in MD lymphoma, miR-126 expression was restored. This strategy demonstrated the role of miR-126 in controlling cell proliferation and targeting the key proto-oncogene *CRK* in GaHV-2 transformed cells.

## 2. Materials and Methods

### 2.1. GaHV-2 Latent Infection in MSB-1 Cell Line and Reagents Used as Reactivation Stimuli

MSB-1 MDCC (Marek’s disease cell culture), a transformed lymphoblastoid cell line harboring GaHV-2 genomes integrated into the cellular genome, was used in this study. It derives from a spleen lymphoma induced by a virulent strain of GaHV-2 (BC-1) and currently serves as a reference [32,33]. It was shown to be co-infected with both GaHV-2 (strain BC-1) and GaHV-3 (strain HPRS24). MSB-1 cells were maintained at 41 °C in 5% CO_2_ in Roswell Park Memorial Institute-1640 (RPMI-1640) medium (Invitrogen^TM^ Life Technologies, Paisley, UK) supplemented with 10% fetal bovine serum, 5% chicken serum, 1% sodium pyruvate (100 mM), 1% nonessential amino acids (Gibco^TM^ Life Technologies, Paisley, UK), 50 µg of streptomycin per ml, and 50 units of penicillin per ml. The MSB-1 cells were treated with either 5 µM 5-azacytidine (5aza) (dissolved in phosphate-buffered saline (PBS)), 3 mM of sodium butyrate (Na butyrate) (dissolved in PBS), or PBS (control). Both reagents were described as reactivation stimuli of GaHV-2 [34]. Cells were recovered 48 h after treatment for RNA extraction.

### 2.2. GaHV-2 Productive Infection in Chicken Embryo Fibroblasts

The chicken embryo fibroblasts (CEFs) were infected with a very virulent strain of GaHV-2 (RB-1B). The primary CEFs were obtained from twelve-day-old chicken embryos treated by trypsinization (Trypsine 10X, Lonza, Basel, Switzerland). Primary CEFs were cultured in Dulbecco’s modified Eagle medium (DMEM) (Invitrogen^TM^ Life Technologies) supplemented with 2.5% fetal bovine serum, 1.25% chicken serum, 50 µg of streptomycin per ml and 50 units of penicillin per ml, 1% fungizone (Gibco^TM^, 250 µg of amphotericin B and 205 µg/mL of sodium deoxycholate), and 1.475 g/L tryptose phosphate broth (TPB) (Sigma, Saint Louis, MO, USA). Four days after the primary CEF culture, these cells were split to give secondary CEFs, which were seeded in a plate (75 cm^2^) with a density of 7× 10^6^ cells. The secondary infected CEFs were cultured in DMEM (Invitrogen^TM^) supplemented with 1% fetal bovine serum, 0.5% chicken serum, 50 µg of streptomycin per ml and 50 units of penicillin per ml, 1% fungizone (Gibco^TM^), and 1.475 g/L TPB (Sigma). Cells were grown at 41 °C with 5% of CO_2_. To infect the cells, the RB-1B bacmid was used. This bacmid consists of the highly virulent RB-1B strain cloned into a bacterial artificial chromosome (bac) [35]. Secondary CEFs were first transfected with an infectious clone of this RB-1B bacmid by lipofection following the manufacturer protocol (Lipofectamine, Invitrogen^TM^). Then, the infection was propagated through cell-to-cell spreading by co-seeding freshly prepared secondary CEFs with previously infected CEFs at a ratio of 3:1.

### 2.3. Ethics Statement of the In Vivo Experiment

All animal work was performed following the appropriate regulations and was approved by the Ethical Commission of CODA CERVA (veterinary and agrochemical research center) in Uccle, Bruxelles (Belgium). Project number 20150216-01.

### 2.4. Experimental Animals

Specific pathogen-free (SPF) B^13^B^13^ white leghorn chickens were obtained from the Institut National de la Recherche pour l’Agriculture, l’Alimentation et l’Environnement (INRAE)—Centre Val de Loire at Tours (France). They were kept in controlled-environment isolators with food and water provided ad libitum in the veterinary and agrochemical research center (CODA-CERVA) at Uccle (Belgium).

### 2.5. Experimental Design

One-day-old chicks were divided into two groups of 12 at hatching. Birds from one group were inoculated (by intramuscular route) at 8 days post-hatching with infectious peripheral blood leukocytes (PBL). These PBL were collected from a RB-1B infected B^13^B^13^ chicken at 42 dpi and stored at −196 °C, as previously described [6,36,37,38]. A quantity of 5 × 10^6^ PBL, corresponding to 1000 plaque forming units (PFU) per ml of cell-associated virus, was used to inoculate the sensitive chicken. The second group served as a mock-infected negative control. They were inoculated with non-infectious cell culture medium. Feather tips were harvested on living animals at 0 and 28 dpi in order to obtain feather tip associated cells, predominantly feather follicle epithelium (FFE). Moreover, at 28 dpi, all birds from each group were humanely euthanized and necropsied for tissue collection. Blood was sampled and sorted to obtain PBL and CD4+ T lymphocytes. Eight organs (brain, cerebellum, lung, heart, spleen, liver, testicle, and kidney) from three uninfected birds were harvested. A portion of each sample was stored in RNAlater^®^ (Thermo Fisher Scientific, Paisley, UK) at −80 °C.

### 2.6. Cell Isolation

PBL were isolated by Ficoll^®^-Paque Plus (GE Healthcare, Chicago, IL, USA) from blood samples of infected and uninfected birds. After blood sampling, 4 mL of whole blood was diluted with PBS to a threefold factor. Fractions of diluted blood were carefully layered onto a Ficoll^®^-Paque Plus cushion and centrifuged at 1400 g (without break) for 40 min at room temperature. PBL were recovered and washed twice in PBS, first at 300 g and then at 500 g. Finally, PBL were resuspended in 1 mL of PBS. Then, 1.10^7^ PBL were sorted to obtain CD4+ T lymphocytes, according to the instruction of the Dynabeads^®^ Sheep anti-Mouse IgG kit (Invitrogen^TM^), using the mouse anti-chicken CD4-UNLB antibody (8210-01, Imtec Diagnostics N.V., Antwerpen, Belgium).

### 2.7. RNA Extraction

Total RNA was isolated by guanidinium thiocyanate-phenol-chloroform extraction (TRI Reagent^®^, Ambion, Vilnius, Lithuania). Thereafter, a DNaseI (NEB) treatment was applied to remove contaminating DNA. RNA was quantified, and its purity was confirmed by the measurement of the A260/A280 ratio with a Nanodrop™ 1000 (Thermo Fisher Scientific). The RNA integrity number was determined by the 2100 Bioanalyzer System™ (Agilent, Santa Clara, CA, USA).

### 2.8. Specific Reverse Transcription (RT)

Specific RT was carried out to measure miRNA expression level following the procedure described by Raymond et al. [39]. Briefly, a premix of 13 µL was prepared, including 500 ng of total RNA, 1 µL of dNTP 10 mM each, and 2 µL of gene specific primer (GSP, 0.5 µM), which hybridized on a specific miRNA and was incubated at 65 °C for 5 min to remove secondary RNA structure. The list of primers used in this study is reported in Table A1. Thereafter, a mix of 7 µL was added to perform the RT. This mix was composed of 4 µL of buffer 5X, 1 µL of DTT 0.1 M, 40 U of RNase inhibitor, and 200 U of the reverse transcriptase SSIII. The samples were incubated at 55 °C for 1 h and at 70 °C for 15 min. In parallel, a reverse transcription with random primers was performed.

### 2.9. Non-Specific Reverse Transcription

To measure reference gene or *CRK* mRNA abundance, an amount of 500 ng or 1 µg of total RNA was reverse transcribed with random hexamers and oligo dT using the iScript™ cDNA synthesis kit (Biorad, Hercules, CA, USA), as recommended by the manufacturer’s protocol (step 1: 25 °C for 5 min; step 2: 42 °C for 30 min; step 3: 85 °C for 5 min). The final cDNAs were diluted 10-fold with nuclease-free water.

### 2.10. Quantitative PCR

Quantitative PCR was performed after the RT (qRT-PCR) using the FastStart Universal SYBR Green Master (Rox) (Sigma-Aldrich, Mannheim, Germany). For the evaluation of miRNA expression, equal amounts of 10 times diluted cDNA (2 µL) were analyzed for each sample in triplicate. In the mix, 2 µL of diluted cDNA, the forward (0.8 µM), and the reverse primers (0.8 µM) were added to obtain a final volume of 10 µL. Relative expression of the reverse transcribed *CRK* messenger RNA was determined by analyzing a dilution series in triplicate by qRT-PCR. To this end, 1.1 µL of diluted cDNA was added to 9.9 µL of premix containing forward and reverse primers at a final concentration of 0.3 µM. The list of primers used in this study is reported in Table A1. PCR cycling parameters were as follows: thermal activation for 3 min at 95 °C and then 40 cycles (denaturation for 30 s at 95 °C, primer annealing for 30 s at 50–60 °C (depending on the target), and extension for 30 s at 72 °C). Dissociation curves were generated post-run to analyze amplicon specificity (55–95 °C, with a heating ramp of 0.1 °C per second).

### 2.11. Reference Gene Selection

To study miR-126 silencing, six reference genes (glyceraldehyde-3-phosphate deshydrogenase (*GAPDH*), β-actin, small nuclear RNA U6 (*U6*), 18S ribosomal RNA (*18S rRNA*), hydroxymethylbilanesynthase (*HMBS*), and succinyldehydrogenase (*SDHA*)) were tested in all the samples. The most stable reference genes in the two experimental conditions (infected versus non-infected) were selected by GeNorm [40]. The study of GaHV-2 tumor cell lines with inducible overexpression of miR-126 and control miRNAs made use of *GAPDH* for gene normalization.

### 2.12. 3′ RACE

3′ RACE was performed using the GeneRacer kit (Invitrogen), according to the manufacturer’s instructions. Primers are listed in Table A1. The obtained products were inserted into a pGEM-T easy vector system (Promega, Madison, WI, USA), and Sanger sequencing was carried out by Eurofins Genomics (Ebersberg, Germany).

### 2.13. DNA Extraction

Cells were recovered (maximum 5 × 10^6^ cells) from cell culture by centrifugation for 5 min at 300 g. The pellet was resuspended in PBS. To extract DNA from the collected organs, approximately 30 mg of tissue was crushed with beads (tissue lyser II) and collected in 100 µL of PBS. Genomic DNA from each sample was isolated with the DNeasy^®^ blood and tissue kit (Qiagen, Hilden, Germany). In order to quantify the amount of DNA and assess the purity of samples, the NanoDrop™1000 (Thermo Fisher Scientific) was used.

### 2.14. Bisulfite Genomic Sequencing Analysis (BGSA)

Bisulfite treatment aims to determine the methylation pattern of DNA regions by converting unmethylated cytosines into uracil; methylated cytosines are not converted. The analysis was performed using an EZ DNA methylation-Gold kit (Zymo Research, Irvine, CA, USA) according to the manufacturer’s instructions. 

### 2.15. Polymerase Chain Reaction (PCR)

Various primers were used in two contexts (Table A1). The first one aimed at determining the transcriptional isoforms of *EGFL-**7***. The next series of PCR aimed at studying the DNA methylation patterns of two CpG island regions located in *EGFL-**7*** after bisulfite treatment. PCR was performed as follows: 94 °C for 5 min, followed by 35 cycles of denaturation (94 °C, 1 min), annealing (55–60 °C depending on the target), and extension (72 °C, 1 min), in a final volume of 25 µL containing five units of Taq DNA polymerase (NEB, New England Biolabs, Ipswich, MA, USA), 0.2 µM of each primer, 0.2 mM of each deoxyribonucleotide, 1.5 mM MgCl_2_, and 250 ng of DNA. PCR products were analyzed using agarose gel electrophoresis. When bisulfite-treated DNAs were subjected to PCR, nested PCR analyses were performed with adapted primers to increase the reaction’s sensitivity.

### 2.16. Cloning and Sequencing of PCR Amplicons

PCR amplicons were either excised from agarose gel or directly extracted using NucleoSpin^®^ Gel and PCR Clean-up kit (Macherey-Nagel™, Düren, Germany). Purified PCR products were ligated into pGEM^®^-T Easy vector system (Promega™, Madison, WI, USA). Competent Escherichia coli (*E. coli*) TG1 were transformed and plated in X-gal lysogeny broth (LB) agar plates using ampicillin as a resistance selection marker (100 µg/mL). Individual colonies were screened for the presence of insert by PCR based on universal primers pair M13 forward–M13 reverse (Table A1). Plasmid DNA was prepared from individual colonies and sequenced using the classical Sanger sequencing approach (Eurofins Genomics, Ebersberg, Germany).

### 2.17. Western Blotting

Ninety-six hours after induction of miRNA expression, MSB-1 cells were harvested and washed with phosphate buffered saline (PBS) at room temperature. Cells were then lysed in a RIPA buffer (150 mM NaCl, 5mM EDTA, 50 mM Tris HCl pH 8.0, 1% NP-40, 0.5% sodium deoxycholate, 0.1% sodium dodecyl sulfate) supplemented with 1 mM phenylmethylsulfonyl fluoride (PMSF), 1X cOmplete^TM^ protease inhibitor cocktail (Roche), and 1% Triton X-100 (Merck). Per lane, 25 µg of protein was resolved by 10% sodium dodecyl sulfate–polyacrylamide electrophoresis gel and actively transferred to a nitrocellulose membrane (Whatman Protran BA85, GE Healthcare Life Sciences, Chicago, IL, USA). The membranes were placed in tris-buffered saline buffer with 0.1% Tween-20 and 5% bovine serum albumin as blocking agent. Primary antibodies used were CRK (610036, BD Transduction Laboratories) and Peptidyl-prolyl isomerase B (PPIB), also called Cyclophilin B (ab16045, Abcam), at 1:1000 and 1:2000 dilutions, respectively. The latter served as an internal control. Following multiple washes, the membranes were probed with horseradish peroxidase-conjugated secondary antibodies at a 1:2000 dilution and incubated for 2 min with SuperSignal West Femto Maximum Sensitivity Substrate or Pierce ECL Western Blotting substrate (Thermo Fischer Scientific) according to the target protein. Proteins were detected by ImageQuant LAS4000 mini (Ge Healthcare Life Sciences), and the relative intensities of the bands were quantified using Image J software (National Institutes of Health, Stapleton, NY, USA).

### 2.18. Plasmid Constructs

The system used in this study is the T-Rex™ system, which is a tetracycline-regulated mammalian expression system that uses regulatory elements from the *E. coli* Tn10 encoded tetracycline (Tet) [41]. The pre-miR sequences corresponding to the miRNA of interest (gga-miR-126) and to control miRNAs (gga-miR-21, gga-miR-155, and MDV1-miR-M7) were inserted into the inducible expression plasmid (pcDNA4/T0) downstream human cytomegalovirus immediate-early (CMV) promoter. The expression vector pcDNA4/T0 and pre-miRNA sequences obtained by PCR (Table A1) were digested with *PmeI* restriction enzyme before ligation. Recombinant constructs were transfected in *E. coli*. Positive clones were subjected to PCR analysis to verify the presence and the orientation of the pre-miR sequences. Plasmid DNAs were purified with the Nucleobond™ Xtra Midi kit (Macherey-Nagel™, Düren, Germany), and the inserts of all vectors were fully sequenced (Eurofins Genomics, Ebersberg, Germany).

### 2.19. Generation of MSB-1 Cells Stably Expressing Pre-miRs Regulated by T-REx™ System

MSB-1 cells were seeded in a 12-well culture plate at a density of 2 million cells/mL, and they were transfected with 2 µg of plasmid DNA. Transfection was performed with the Nucleofector™ Amaxa device, and the selected program was X001. First, cells were mixed to 100 µL of nucleofector reagent (kitT, LONZA, Basel, Switzerland), then the plasmid DNA was added to the mixture. After cells were transfected, 500 µL of media was added into the cuvette. The transfected cells were recovered and put in a well of a 12-well plate and maintained for 24 h in the cell incubator. The next day, cells were washed with a complete medium without antibiotics. Forty-eight hours after transfection, cells were treated with antibiotics to begin cell selection (12.5 µg/mL of blasticidin and 75 µg/mL of zeocin). The pcDNA6/TR plasmid was the first plasmid to be transfected into the MSB-1 cells. After 4 weeks of selection with blasticidin, the second plasmid (pcDNA4/T0) was introduced. A second selection was made with the combination of blasticidin and zeocin for 4 weeks. Pre-miRNAs’ expression was induced by treating cells with tetracycline (1 µg/mL). The expression levels before and after the induction were assessed by qRT-PCR after specific reverse transcription of extracted RNA, as described above.

### 2.20. MTS Cell Proliferation Assay

Here, 75000 cells per ml of each of the established cell lines were seeded in a six-well plate and treated with tetracycline for 96 h. To investigate cell viability, 20 µL of the tetrazolium dye MTS (3-(4,5-dimethylthiazol-2-yl)-5-(3-carboxymethoxyphenyl)-2-(4-sulfophenyl)-2H-tetrazolium) solution (Abcam) was added to 200 µL of each sample in a 96-well plate. The cellular metabolism reduces the tetrazolium dye to purple colored formazan. The plate was incubated for 2 h. The absorbance, reflecting the cellular metabolic activity and thus cell viability, was then recorded at 490 nm using a Thermo electron corporation Multiskan Ex spectrophotometer.

### 2.21. Statistical Analysis

All data are presented as mean and analyzed by analysis of variance (ANOVA) or Student’s *t*-test with the R software and Excel. Data were considered significant at *p* < 0.05.

## 3. Results

### 3.1. MiR-126 Is Repressed in the Target Cells of GaHV-2 Latency and Transformation

MiR-126 expression pattern was measured by qRT-PCR in a GaHV-2 transformed tumor cell line (MSB-1) and cells derived from eight organs collected from non-infected birds (brain, cerebellum, lungs, heart, spleen, liver, testicle, and kidney). Then, miR-126 was quantified in several in vitro and in vivo samples to monitor its expression level at key steps of the viral life cycle.

**Figure 1:**
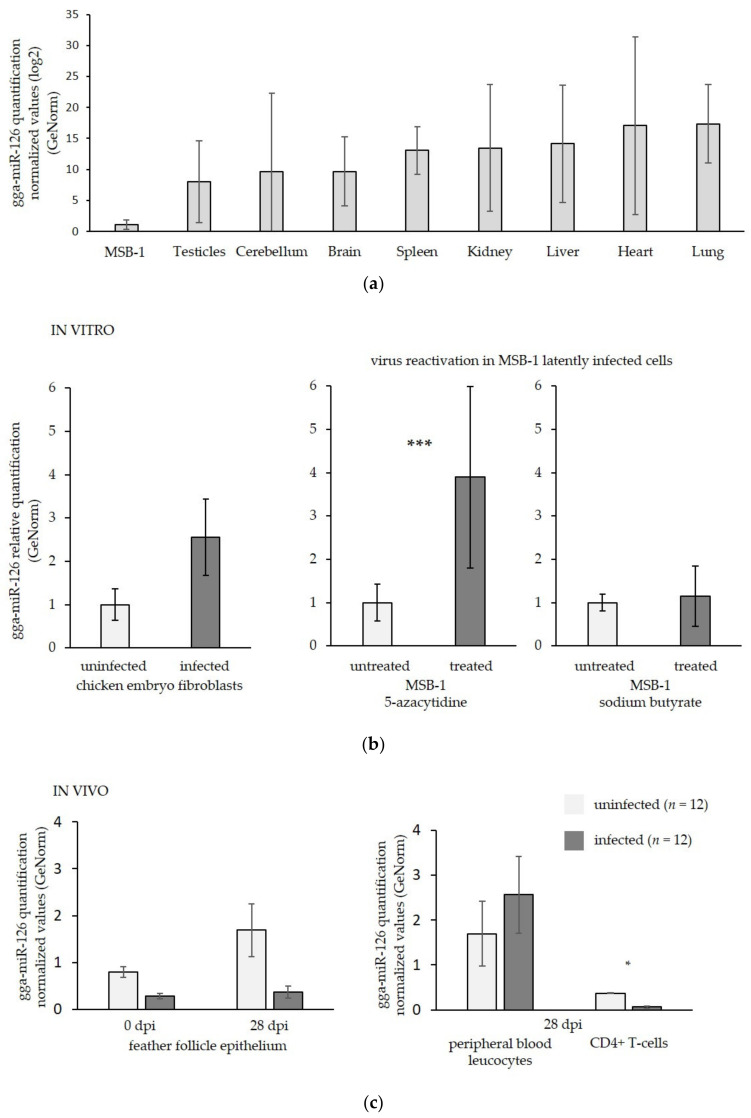
MiR-126 quantification in in vitro and in vivo samples. (**a**) Quantification of miR-126 in eight organs (brain, cerebellum, heart, lung, spleen, liver, testicle, and kidney) from three uninfected chicken and in a MSB-1 cell line by qRT-PCR; (**b**) quantification of miR-126 in in vitro samples representing the different phases of the viral life cycle of GaHV-2 (two independent assays with technical triplicates); (**c**) quantification of miR-126 in in vivo samples. Uninfected or untreated samples are represented in light grey. Infected or treated samples are represented in dark grey. In all the graphs, the X-axis represents the samples used for quantification, and the Y-axis represents normalized values with three reference genes (*GAPDH, HMBS, 18S rRNA*) selected by GeNorm software. Student’s *t*-test: *, *p* < 0.05; ***, *p* < 0.001 represent significant results. Error bars are standard deviations.

Near extinguished miR-126 expression was observed in GaHV-2 tumor cells compared with the expression level detected in cells obtained from eight organs of uninfected chicken (Figure 1a). The highest expression was measured in highly vascularized organs with an expression yield up to 30,000-fold higher than that observed in the GaHV-2 transformed cell line.

In order to assess miR-126 expression in the most relevant conditions and in the cell contexts corresponding to the crucial steps of GaHV-2 infection, different in vitro and in vivo samples were analyzed. First, CEFs supporting the in vitro replicative viral phase were tested before and after infection with the RB-1B very virulent GaHV-2 strain. 

Replicative infection induced a slight increase, but no significant change in miR-126 expression, which was lowly expressed even in the non-infected cells (Figure 1b). Then, MSB-1 cells were used to compare miR-126 expression during latency (untreated) and upon reactivation. To induce reactivation, transformed cells were treated with either a DNA methyltransferase inhibitor (5aza) or a histone deacetylase inhibitor (Na butyrate) (Figure 1b). GaHV-2 reactivation was tested by qRT-PCR, which demonstrated the overexpression of late genes encoding structural (VP5) and non-structural (pp38) proteins (data not shown). GaHV-3 reactivation was not investigated. After reactivation of GaHV-2 with the 5aza, a significant induction, characterized by a fourfold increase of miR-126 expression, was observed (Figure 1b). No significant change was detected after treatment of the GaHV-2 transformed cells with the Na butyrate (Figure 1b). These results suggest a potential implication of DNA methylation on the modulation of miR-126 expression, while no impact of histone post-translational modification was observed.

To test the effect of GaHV-2 infection on miR-126 expression in vivo, specific target cells of the GaHV-2 life cycle were analyzed. Feather follicles were harvested as they support the late GaHV-2 replicative phase. As observed in in vitro infected cells, no significant change was induced during the GaHV-2 productive infection (Figure 1c). PBL and sorted CD4+ T cells isolated from infected and non-infected birds were tested to see whether the target cells of GaHV-2 latency and transformation show variations in miR-126 expression (Figure 1c). At day 28 pi, no significant change in miR-126 expression was noticed in PBL, while significant repression (fivefold) of miR-126 was quantified in CD4+ T lymphocytes from tumor tissue of infected chicken when they were compared with CD4+ T lymphocytes sorted from uninfected chicken (Figure 1c). These results show that miR-126 is repressed during the tumorigenesis phase of GaHV-2. This repression is specifically observed in the target cells of the latent infection in vivo. As this downregulation might be driven by DNA methylation, this epigenetic mark was further investigated.

### 3.2. Epidermal Growth Factor Like 7 (EGFL-7) Transcripts Analysis

**Figure 2:**
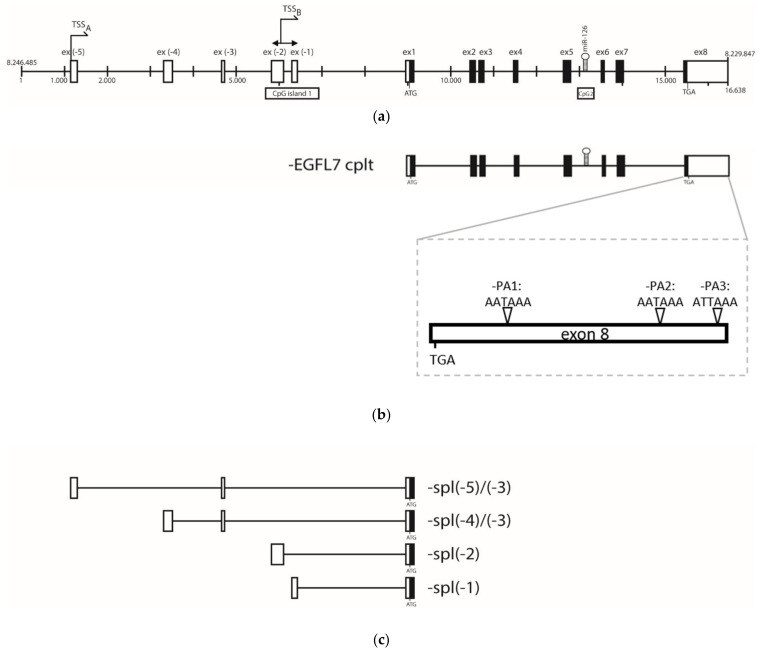
Schematic representation of the epidermal growth factor like-7 (*EGFL-7*) gene and alternative transcripts. (**a**) The full-length *EGFL-7* gene with two major transcriptional start sites (TSSs) and CpG islands. Gene structure and exon annotations were established from bioinformatics predictions available for *EGFL-7*. (**b**) An alternative transcript including exons 1 to 8 and a detail of exon 8 showing the different functional polyadenylation signals (PA) sites (PA1, PA2, and PA3). TGA is the codon stop. Primers used to perform the 3′ RACE are listed in Table A1. (**c**) Four alternative transcripts from exon −5 to exon 1. All alternative transcripts are available in Figure S1. Non-coding exons are represented by white boxes. Coding exons are represented by black boxes. The black bars are introns. MiR-126 is represented by a stem-loop structure in the fifth intron.

The repression of miR-126 observed in CD4+ T lymphocytes obtained from tumors of infected chicken raised the question of whether miR-126 expression is controlled by its host gene promoter or its own promoter. To answer this question, transcriptional start sites (TSSs) and 3′ ends along the *EGFL-7* gene were determined as *EGFL-7* transcription has not yet been characterized in chicken. In Figure 2a, the chicken *EGFL-7* gene structure is represented with all the potential exons existing in the different isoforms. The 3′ terminus of the *EGFL-7* transcript was determined by 3′ RACE (rapid amplification of cDNA ends) analysis (primers are listed in Table A1). Three functional polyadenylation signals were identified within the exon hosting the stop codon of *EGFL-7*. Thereby, exon 8 is defined as the terminating exon, creating three 3′ UTR isoforms ranging from 268 to 1004 nucleotides (Figure 2b). The 5′ end was mapped thanks to a published database created by CAGE (cap analysis of gene expression) analysis of transcripts collected from chicken embryos during early development steps [42]. This analysis revealed alternative transcriptional start sites (TSSs) associated with five putative exons localized upstream the exon hosting the start codon of the EGFL7 protein (Figure 2c). The main TSSs were mapped: the well-defined TSS_A_ and dispersed TSS_B_ (Figure 2a). Based on this gene structure, *EGFL-7* transcript isoforms were determined by RT-PCR. Splicing patterns showed a complex alternative transcription (Figure S1). Altogether, miR-126 was intimately associated with the intron localized between the fifth and the sixth exon of *EGFL-7*. In addition, the *EGFL-7* gene was further analyzed to identify CpG islands using the in silico method described by Gardiner–Garden and Frommer, 1987 [43]. Strikingly, one CpG island was associated with TSS_B_, which had been identified as the major TSS in the CAGE analysis. This CpG island (named CpG1, Figure 2a) appears to be conserved when human and chicken *EGFL-7* gene structure is compared and was shown to be crucial for controlling miR-126 expression in humans [26]. A second CpG island (CpG2) was localized near the premiR-126 sequence (Figure 2a). Based on these sequence analyses, DNA methylation patterns were established and compared with the miR-126 expression profile.

### 3.3. Low Level of miR-126 Expression Is Associated with DNA Hypermethylation of EGFL-7 CpG Islands in the Target Cells of Latent GaHV-2 Infection

In the first series of experiments, miR-126 expression was shown to be nearly extinguished in tumor CD4+ T cells infected with GaHV-2. As the DNA methyltransferase inhibitor released this inhibition and CpG islands were found in the vicinity of TSSs of a large variety of transcripts possessing pre-miR-126 in one of their introns, we investigated patterns and levels of DNA methylation within the CpG1 and CpG2 regions of the *EGFL-7* gene in the most relevant conditions: in tumoral MSB-1 cells and in samples obtained from uninfected and infected birds. Subsequently, we examined whether a correlation could be established between the DNA methylation pattern and the miR-126 expression at the different stages of the infection.

**Figure 3:**
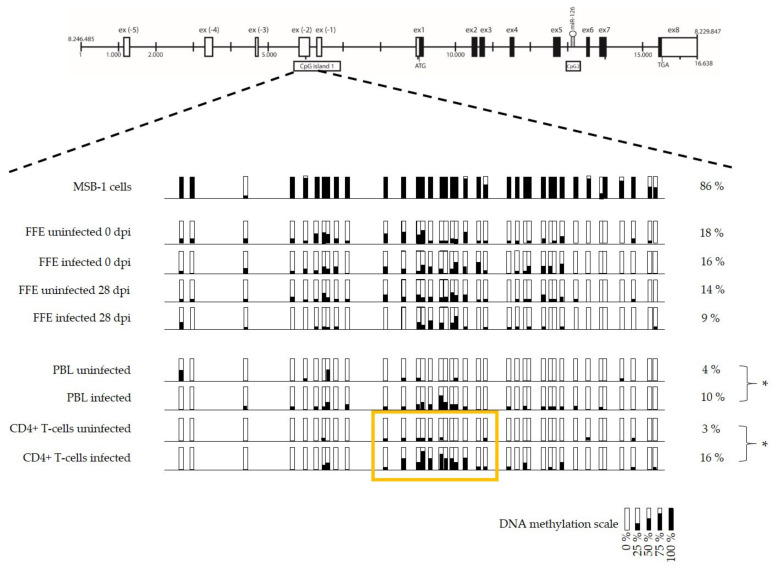
DNA methylation pattern of the CpG1 region in the MSB-1 cell line and in vivo samples from infected and uninfected birds. The horizontal line represents introns. The percentage of methylation is indicated by the black coloration inside the rectangles that represent each cytosine/guanine dinucleotide at a precise location. The yellow frame surrounds a region with a differential DNA methylation pattern. Two-way analysis of variance (ANOVA): *, *p* < 0.05 represent significant results.

**Figure 4:**
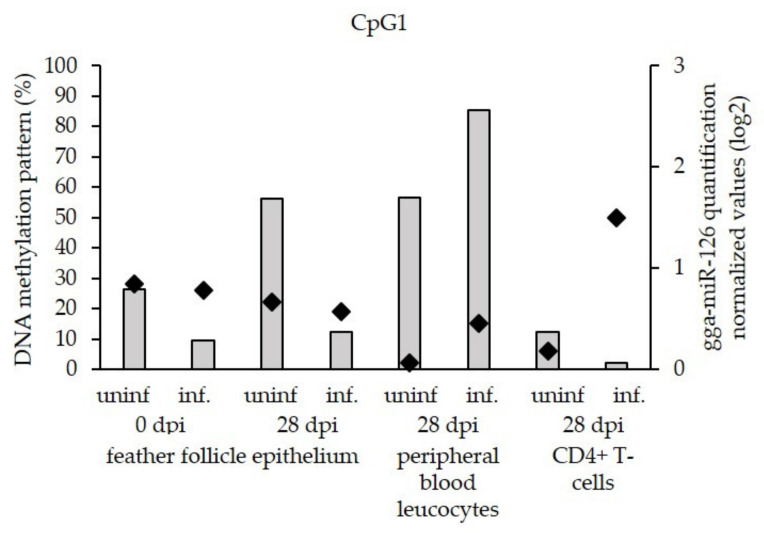
Correlation between the miR-126 quantification (right Y-axis, gray histograms) and the DNA methylation pattern in CpG1 (left Y-axis, black diamonds). The DNA methylation percentage is one of the yellow frame regions of Figure 3. The X-axis represents the different samples used: feather follicle epithelium at 0 and 28 days dpi, as well as peripheral blood leucocytes and CD4+ T lymphocytes from either uninfected (uninf.) or infected (inf.) birds at 28 dpi.

The DNA methylation pattern was established in CpG1 by detailing modifications observed at each CpG dinucleotide site in MSB-1 cells and feather follicle epithelium, peripheral blood leucocytes, and CD4+ T-cells obtained from uninfected and infected birds (Figure 3). The highest global DNA methylation percentage was observed in the MSB-1 cells with 86% of methylation compared with the in vivo samples that displayed methylation levels ranging from 3 to 18%. The methylation pattern did not significantly differ between infected (at 28 dpi) and uninfected feather follicle epithelium, target cells of the productive viral phase, nor could it be related to the miR-126 expression in these cells. However, the in vivo experiment showed a significant increase in methylation level in latently infected leucocytes (10% in PBL and 16% CD4+ T cells) compared with their uninfected homologues (4% and 3%, respectively). A correlation could be observed between this hypermethylation during the viral tumorigenesis phase and the repression of miR-126 (Figure 4). The CD4+ T samples even displayed a highly discriminative DNA methylation pattern in a central motif of 12 CpG nucleotides. Whereas the methylation levels in this region reached 6% in the uninfected group, it was as high as 50% in the infected group. Moreover, the low DNA methylation percentage (6%) was associated with a high expression level of miR-126 in the uninfected group. In the infected group, the high percentage of methylation (50%) corresponded to a low expression level of miR-126 (Figure 4).

**Figure 5:**
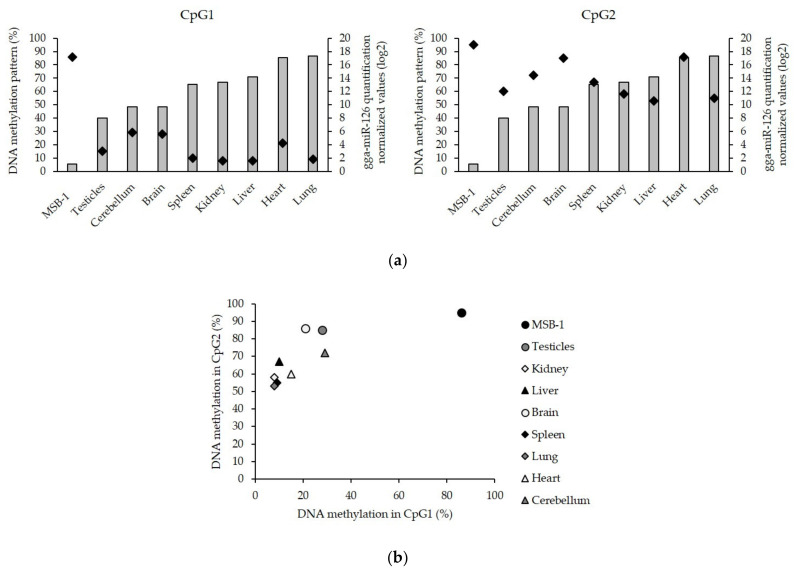
Comparison of the DNA methylation pattern in the CpG1 and CpG2 regions of *EGFL-7*. (**a**) Correlation between the miR-126 quantification (right Y-axis, gray histograms) and the DNA methylation percentage (left Y-axis, black diamond) in the CpG1 and CpG2 regions of different samples used (MSB-1 cell line and the eight organs from three uninfected chicken). (**b**) Comparison of the DNA methylation percentage in the CpG1 region (X-axis) and CpG2 region (Y-axis).

In the above-described CpG1, the percentage of DNA methylation was higher in the MSB-1 cells (86%) in comparison with the cells isolated from eight organs of uninfected animals (from 8% to 29%) (*p* < 0.001) (Figure 5a). The detected hypermethylation in MSB-1 cells was shown to be correlated with a low miR-126 expression, whereas the lower level of methylation found in the other tissues corresponded to a higher miR-126 expression (Figure 5a). The second CpG island, located around the pre-miR-126 sequence, was also examined. Methylation levels in this region did not differ significantly, although the maximum value was observed in the MSB-1 cells (95%). No correlation was established in the CpG2 island between the global DNA methylation levels and miR-126 expression (Figure 5a). A combined view of the DNA methylation status of these two regions revealed GaHV-2 tumor cells as outsiders as both CpG islands were hypermethylated (Figure 5b).

These results further suggest that miR-126 repression in GaHV-2 target cells of latency and transformation is linked to DNA hypermethylation set in the *EGFL-7* CpG1 island. In order to investigate the consequences of miR-126 repression in the GaHV-2 tumorigenesis, a functional assay was carried out to restore miR-126 expression.

### 3.4. Generation of GaHV-2 Tumor Cell Lines with Inducible Overexpression of miR-126 and Control miRNAs

In order to test the biological effects induced by miR-126 silencing during MD lymphoma development, a strategy was developed to restore miR-126 expression in transformed CD4+ T cells propagated from MD lymphoma (MSB-1). After transformation, tumor cells easily propagate in vitro and keep several features of the cancerous phenotype; however, they are refractory to another viral infection. Therefore, we employed a conditional expression system (Tet-on inducible expression plasmids after selection and integration) that was recently used to modify gene expression in the GaHV-2 transformed cells propagated in vitro [44]. Stable cell lines were established to allow the inducible expression of the miRNA of interest (gga-miR-126) and control miRNAs (gga-miR-155, gga-miR-21, and MDV1-miR-M7). In the MD context, gga-miR-21 and MDV1-miR-M7 are overexpressed [6], while gga-miR-155 was shown to be repressed [37]. A viral functional ortholog of the latter is produced during viral infection [45].

MSB-1 cells were successively transfected with two plasmids and selected by antibiotic resistance. The first transfected plasmid expresses the tetR repressor (pcDNA6/TR). The second contains the different pre-miRNA sequences (pcDNA4/T0). In the absence of tetracycline, miRNA expression is inhibited by the repressor (TetR). The addition of tetracycline to the culture medium relieves this repression, leading to the activation of the miRNA expression.

First, the expression of tetR was evaluated by qRT-PCR in the different cell lines treated or not with tetracycline (primers are listed in Table A1). The repressor should be constitutively expressed in the stable cell lines that integrated the plasmid of interest (pcDNA6/TR). Similar amounts of tetR transcript were detected in the different samples (Figure S2a). This result confirms the constitutive overexpression of this repressor in all of the established cell lines, independently of the presence of tetracycline. Secondly, the expression of the different miRNAs in their respective stable cell lines was assessed after a 48 h treatment with tetracycline. As expected, miR-126 and miR-155 were repressed in treated cells containing an empty vector. On the other hand, tetracycline highly increased the miR-126 and miR-155 expression by the cell lines that included the corresponding plasmids (Figure S2b). A lesser increase of miR-21 and MDV1-miR-M7 was observed compared with the basal level in the stable cell line containing the empty plasmid, which is in agreement with previous data showing high constitutive expression of these two miRNA in MSB-1 cells. Tetracycline is thus able to trigger the overexpression of miR-126 and in the controls miR-21 and miR-155.

### 3.5. Cell Proliferation Is Impaired Following miR-126 Overexpression

**Figure 6:**
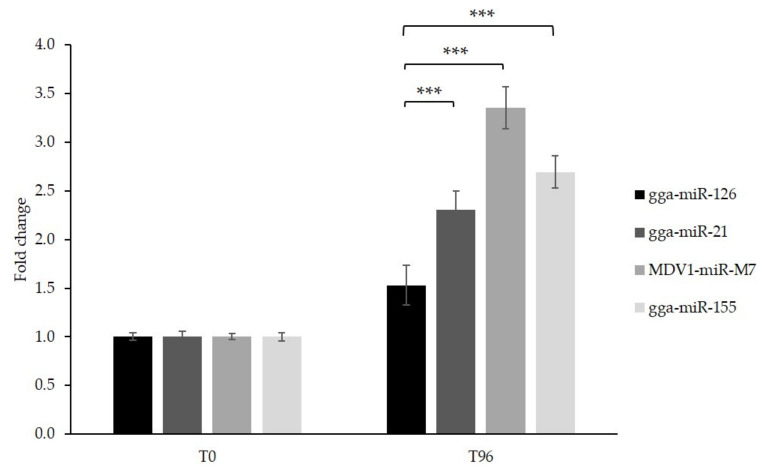
Cell proliferation after tetracycline induction at two time points. Cell proliferation evaluation by MTS assay, before (T0) and 96 h (T96) after induction of the expression of miR-126 and control miRNAs in stable MSB-1 cell lines containing the respective plasmids, presented as the mean fold change (three independent assays with technical triplicates) in cell viability between the two time points. Student *t*-test: ***, *p* < 0.001 represent significant results. Error bars are standard deviations of triplicates.

The proliferation of GaHV-2 tumor cells was evaluated by a colorimetric MTS assay before and 96 h after induction of miR-126 and control miRNA overexpression. The restoration of miR-126 obtained in this way caused a significant reduction in cell proliferation (*p* < 0.001) compared with the treated control cell lines. The experiment was repeated in three independent assays, and similar results were obtained (Figure 6).

The cell proliferation increased 2.30 to 3.35 times in the cultures overexpressing an oncogenic control miRNA, whereas cells expressing miR-126 multiplied by a factor of 1.5. These data indicate that, even though cell division still occurred at a low level, miR-126 overexpression induces a slowdown in cell proliferation.

### 3.6. The Proto-Oncogene CRK Is Targeted and Down-Regulated by miR-126 in GaHV-2 Transformed Cells

**Figure 7:**
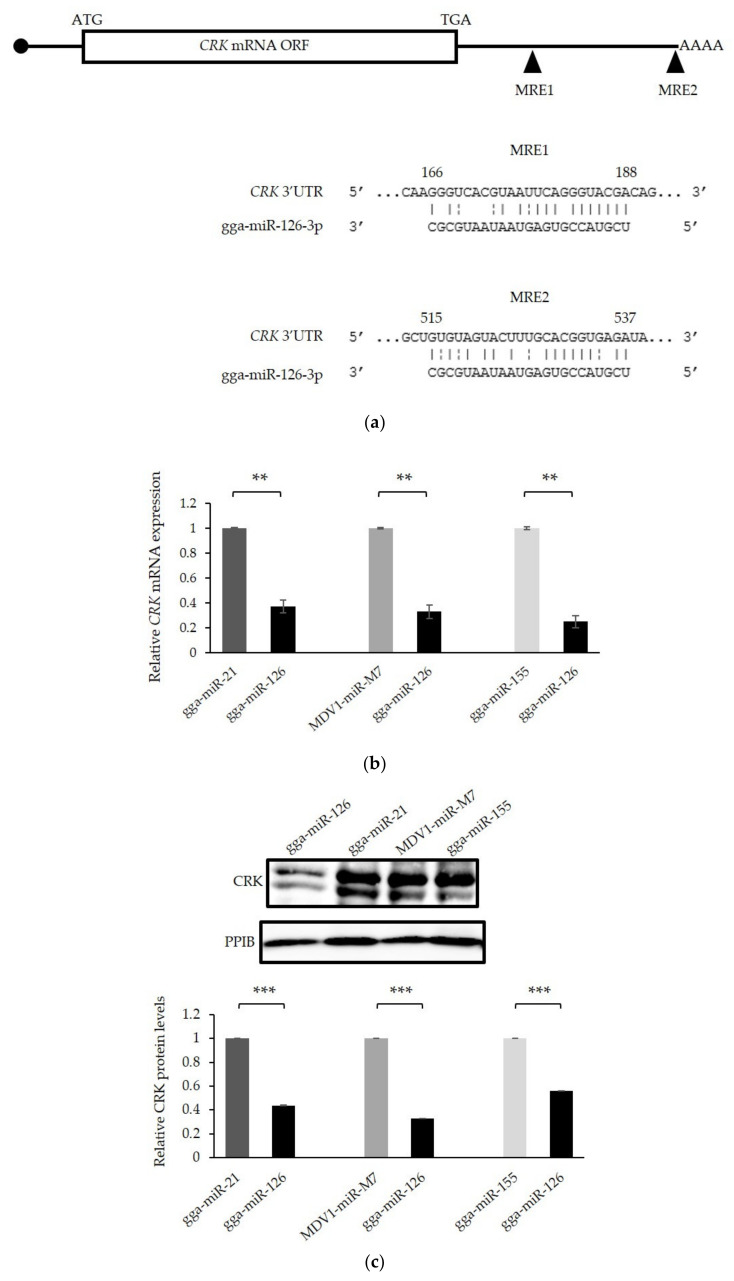
Effect of miR-126 restoration on *CRK* expression levels. (**a**) Location of miRNA response elements (MREs) for miR-126-3p, indicated by the black triangles, in the 3′ UTR of gallid *CRK* mRNA. (**b**) Relative quantification of *CRK* mRNA levels by qRT-PCR 96 h (T96) after induction of the expression of miR-126 and control miRNAs in stable MSB-1 cell lines containing the respective plasmids. (**c**) Western blot analysis and relative quantification, in arbitrary units, of the CRK protein levels produced by the different stable cell lines at T96. Student’s *t*-test: **, *p* < 0.01; ***, *p* < 0.001 represent significant results. Error bars are standard deviations of technical triplicates.

CT10 regulator of kinase (CRK) is a ubiquitous adaptor protein in signaling pathways implicated in cell adhesion, proliferation, and migration [46]. It was found to be directly targeted and downregulated by miR-126 in humans. In several human malignancies, repression of miR-126 was associated with elevated levels of CRK, leading to enhanced tumor development and metastasis [25,46,47,48,49,50,51,52]. The structure of the gallid cellular CRK protein is closely related to human CRKII. MiR-126 target sites in the 3′ untranslated region of gallid *CRK* are shown in Figure 7a. The relative expression of *CRK* messenger RNA at 96 h after tetracycline treatment was determined by qRT-PCR. It revealed a 2.7- to 4-fold decrease of *CRK* mRNA levels in cells overexpressing miR-126 compared with the tested controls (Figure 7b) (*p* < 0.001), suggesting a degradation of the target mRNA. On the Western blot, the CRK band is detected at approximately 38 kDa and appears as a doublet [47]. Western blotting confirms significant downregulation (1.8- to 3.1-fold, *p* < 0.001) of CRK protein levels in both bands of the sample extracted from the cell line expressing miR-126. (Figure 7c). In this study, CRK appeared to be downregulated by a reduction of mRNA, whereas others reported *CRK* mRNA levels to remain stable while CRK protein levels are diminished by miR-126 overexpression [25,46,49,53].

## 4. Discussion

Apart from being a major disease affecting poultry health, MD is a valuable natural model for rapid-onset herpesvirus-induced T-cell lymphomas [13]. During GaHV-2 infection, several cellular miRNAs were shown to be misexpressed along with the virus-induced tumorigenesis [6,54,55]. Currently, little is known about the molecular mechanism by which cellular miRNAs modulate GaHV-2 pathogenesis and the behavior of the host cell during viral infection. The importance of cellular miRNAs in host–pathogen interactions has been highlighted recently in humans and animals [5,7,17]. In humans, for example, miR-138 was shown to mediate human cytomegalovirus (HCMV)-induced angiogenic response by the inhibition of SIRT1, which is an inhibitor of a pro-angiogenic regulator (p-STAT3) [17]. During MDV infection, repression of miR-26a and overexpression of miR-221/222 were shown to have an impact on immunity (interleukin-2) and cell cycle progress (p27kip1), respectively. These misregulations contributed to the induction or maintenance of the tumorigenesis process.

This study focused on a cellular miRNA, miR-126, mediating proper angiogenesis and being identified in several cancers as a tumor suppressor gene whose expression is lost during cellular transformation. Our study investigated its expression along key steps of the viral infection in different cell types involved in Marek’s disease pathogenesis. Major findings obtained in this study include that miR-126 expression was nearly extinguished in GaHV-2-transformed CD4+ T-cells (MSB-1), both in vitro and in vivo, when compared with cells obtained from different organs of uninfected chickens or with cells harboring the replicative phase of the infection. The repression of miR-126 in latently infected leucocytes was shown to be mediated by DNA hypermethylation of its gene promoter. In addition, miR-126 restoration in an MD lymphoma cell line significantly reduced cell proliferation, with *CRK* being a potential target.

Our first data showed miR-126 to be highly expressed in the liver, heart, and lungs, while a very low expression of this miRNA was measured in CD4+ T transformed cell lines (MSB-1 cells). Other studies quantified this miRNA in various organs in humans and mice. An elevated expression of miR-126 was observed in highly vascularized organs such as the heart, spleen, testis, lungs, and liver, which is in agreement with our data [22,56,57]. We found miR-126 to be repressed at crucial steps of the GaHV-2 infection in vitro and in vivo. Several studies previously suggested this repression in the MD context, however, none of them investigated the miR-126 expression in such a large panel of samples. A low expression of miR-126 was noted in vitro in several transformed cell lines derived from tumors induced by different GaHV-2 viral strains (RB-1B, BC-1, and GA strains) [54]. In vivo, the repression was formerly observed in GaHV-2-induced lymphoma in the liver compared with normal liver tissue and non-sorted lymphocytes obtained from non-infected chicken [58]. Two additional reports showed repression of miR-126 in non-sorted PBL from two susceptible and infected chicken lines (B13/B13 and 7_2_) [6,55]. Moreover, a significantly reduced expression of miR-126 was observed in the susceptible 7_2_ line chickens infected with a very virulent strain compared with infected 6_3_ line chickens that are MD-resistant [51]. Furthermore, this miRNA was found to be repressed in numerous human cancers, giving its potential role as a tumor suppressor [19]. The originality of the present study is to monitor the miR-126 expression level and, additionally, to establish its correlation with the associated DNA methylation pattern of its host gene.

In humans, the majority of miRNAs are localized in intronic regions into a coding or non-coding transcriptional unit [59]. These intronic miRNAs are usually positioned in the same orientation as their host genes and controlled by the promoters driving the primary mRNA transcripts [59]. Intronic miRNAs and their host genes are thus most likely co-regulated and generated from a common transcript precursor [60]. MiR-126 is localized in the fifth intron of the epidermal growth factor like-7 (*EGFL-7*) host gene. The presence of alternative promoters during chicken development was highlighted by sorting and analyzing data launched by Lizio and collaborators [42]. It revealed the existence of the main initiation site controlled by a CpG island. Analysis of the alternative transcripts showed the production of different variants of the EGFL7 protein using a cassette exon (ex4 and ex5), intron retention (-delta-int2), and cryptic 3′ splice site (-Var-int6) mechanisms (Figure S1).

In contrast, all alternative transcripts correspond to potential isoforms of pri-miRNA, whose processing may lead to miR-126 expression. Three alternative isoforms, transcribed from different promoters, were identified in human [26]: a long transcript (S1) corresponding to the entire gene sequence, an alternative transcript (S2) (from an alternative exon in the second intron), and a short transcript (S3) beginning in the exon 7 upstream pre-miR-126. The alternative transcript (S2) of *EGFL-7* was the primary form from which pre-miR-126 was processed [26]. The role of each transcript on miRNA processing and expression in chicken remains unclear.

During infection, DNA viruses hijack several cellular processes that are essential either to their replication or maintenance. For instance, they use cellular factors to initiate their own transcription or to ensure their replication, as well as epigenetic factors to modulate their gene expression [61]. In addition, viral infections were shown to affect the epigenetic state of host genes, leading to the development of tumors in humans [62,63]. For example, in primary effusion lymphoma (PEL), caused by Kaposi’s sarcoma associated herpesvirus (HHV-8), de novo DNA methyltransferase (DNMT3a) is recruited to chromatin by the viral protein LANA. This leads to the hypermethylation of *p16INK4a* [64,65]. A similar mechanism has been observed during Epstein Barr virus (HHV-4) infection. E-cadherin was repressed owing to the activation of de novo DNMTs by latent membrane protein 1 (LMP1) [65]. In this report, repression of miR-126 was associated with DNA hypermethylation in the first CpG island of its host gene. This phenomenon is in agreement with previous studies reporting the methylation status of the corresponding CpG island localized in the human *EGFL-7* gene in different cancer cell types [26,28,29,66]. Additional controls might have been used to establish the DNA methylation pattern during GaHV-2 infection, such as fresh tumor cells from infected chicken. However, cells collected from organ samples contain a mixed cell population. In this mixed population, it would be difficult to assign a methylation status to a cell type in particular, as the specific target cells of latency and transformation. This status can in fact vary from one cell type to another and the global methylation status of a mixed cell population might mask the effect of CD4+ T-cells.

As mentioned earlier, viral infections may lead to misregulation in the expression of the host miRNA repertoire. They may be up- or downregulated. In humans, aberrant expression of miRNAs has been reported to have an impact on carcinogenesis [19]. We evaluated cell proliferation through a colorimetric MTS assay, before and 96 h after inducing the expression of miR-126. The restoration of miR-126 in CD4+ T lymphoma cell lines was shown to significantly reduce cell proliferation compared with the tested controls. Even if some interference cannot be ruled out regarding the use of specific miRNA controls, miR-126 significantly reduced cell proliferation when compared with each control independently. The control miRNAs used in this study substitute the scrambled short hairpin RNA controls that are used when the functionality of a candidate miRNA is studied [67,68]. Feng et al. demonstrated that overexpression of miR-126 in human gastric cancer cell lines (SGC-7901) induces cell cycle arrest at G0/G1 phase [46]. In addition, this overexpression was associated with an inhibition of migration and invasion of gastric cancer cell lines. Inhibition of cell proliferation was also observed in hepatocellular carcinoma cells owing to the activation of apoptosis [69]. MiR-126 functionality was studied in numerous human cancers and brought evidence that this miRNA is implicated in several biological cell processes such as apoptosis, cell invasion, cell migration, cell cycle, and lipid metabolism (miR-126 functionality is reviewed in Ebrahimi et al., 2014 [19]). Several targets of miR-126 were validated in this context. Nevertheless, miR-126 seems to have a more complicated behavior than only a tumor suppressor gene, and its function might not be dependent on one target gene, but instead on competition or balance among these target genes for a specific type of cancer [48,49,70].

The cellular homolog of the transforming viral sarcoma virus CT10 regulator of kinase oncogene (c-*CRK*) was found to be overexpressed in multiple human malignancies. CRK constitutes a central adaptor molecule in signaling pathways controlling cell adhesion, proliferation, migration, and invasion [46]. It consists of three domains. The amino-terminal Src Homology 2 (SH2) domain interacts with phosphorylated tyrosine motifs engaged mainly by oncogenic tyrosine kinases or integrin activation [71]. The extracellular signal is propagated to downstream cellular effectors by binding of proline-rich ligands by the N-terminal Src Homology 3 (SH3N) domain. The C-terminal Src Homology 3 (SH3C) domain modulates this binding’s affinity [72]. Many downstream binding partners have been identified and include guanine nucleotide exchange factor proteins, SOS1, C3G, and DOCK180. Their interaction with CRK leads to the activation of small GTPases Ras, Rap1, and Rac1 that exert diverse roles in cell motility and growth [71]. An elevation of CRK protein levels has been associated with the transforming activity in several human cancers [71]. The protein was shown to be directly targeted and downregulated by miR-126 in different cell types, and deregulation of this axis has been observed in human tumors such as gastric carcinoma [46,48,51], pancreatic duct adenocarcinoma [52], mammary carcinoma [49], and melanoma [47]. An example highlighting the effect on tumoral growth mediated by the miR-126/CRK axis in humans concerns non-small cell lung carcinoma [25,50]. MiR-126 expression was found to be reduced in this cell line. Its overexpression led to a decrease of CRK levels and repression of the tumor development. Cell proliferation was decreased by an arrest of the cell cycle at the G0/G1 stage. The CRK family proteins were also attributed with a role in the selective regulation of chemokine-dependent adhesion, migration, and diapedesis of T cells to effector sites [73]. In our study, restoration of miR-126 expression resulted in post-transcriptional downregulation of *CRK*, decreasing the *CRK* mRNA and protein levels. Our data indicate that *CRK* is a target gene of miR-126 and that its downregulation by miR-126 might be, at least partially, responsible for the observed effect on cell proliferation. As overexpression of miR-126 does not induce an arrest of the cell proliferation in this transformed CD4+ T cell line, it is likely that other factors play a role in cell transformation. Further experiments are needed in order to investigate whether additional miR-126 target genes are implicated in the noted cell proliferation decline.

## 5. Conclusions

Taken altogether, these data show a potential tumor suppressor role of miR-126 in the context of Marek’s disease lymphoma. The repression of this miRNA during latency and tumorigenesis was confirmed and was demonstrated to be due to the hypermethylation of its gene promoter. Inducible overexpression of miR-126 in a transformed cell line was associated with significant repression of cell proliferation. Our data suggest *CRK* to be a potential target of miR-126 and to mediate, at least partly, the observed reduction of cell proliferation. However, the study of other potential miR-126 targets would bring a more precise answer on which pathways miR-126 silencing has an impact on during virus-induced lymphomagenesis.

## Data Availability

All relevant data are within the paper and its Supplementary Materials.

## Supplementary Materials

The following are available online at https://www.mdpi.com/article/10.3390/microorganisms9061339/s1, Figure S1: Schematic representation of all transcripts encoding the epidermal growth factor like-7 (*EGFL-7*) gene; Figure S2: Quantification of the tet repressor and different micro-RNAs in the stable MSB-1 cell lines treated with tetracycline.

## Appendix A

**Table A1:** Information for primers. Gallus gallus (gga), Gallid alphaherpesvirus-2 (GaHV-2), gene-specific primer (GSP), locked nucleic acid (LNA), universal primer (UP), forward primer (Fwd), reverse primer (Rev), CpGi means CpG island. When nested PCR was performed, Fwd and Rev primers were numbered: number 1 means the primer was used for the first PCR reaction and number 2 means the primer was used for the nested PCR reaction. R stands for purine, a G or A nucleotide. Y stands for pyrimidine, a C or T nucleotide. Bold and italic letters are the restriction site of *Pme*I restriction enzyme.

**Primers of qRT-PCR**		
**Primers Name**	**Sequences (5′–3′)**	**GenBank Accession No.**
gga-miR-126-3p	GSP: CATGATCAGCTGGGCCAAGAGCGCATTATT LNA: TCGTACCGTGAGTA UP: CATGATCAGCTGGGCCAAGA	
*SDHA*	Fwd: TTCCCGTTTTGCCTACGGTG Rev: CTGCCTCGCCACAAGCATAT	XM 419054.2
*U6*	Fwd: CTCGCTTCGGCAGCACATATAC Rev: TTTGCGTGTCATCCTTGCGC	(Zhao et al., 2015 [69])
*18S rRNA*	Fwd: GGCGGCTTTGGTGACTCTAG Rev: ATCGAACCCTGATTCCCCGT	AF173612.1
*HMBS*	Fwd: GGCTGGGAGAATCGCATAGG Rev: TCCTGCAGGGCAGATACCAT	XM 417846.2
*GAPDH*	Fwd: GTCCTCTCTGGCAAAGTCCAAG Rev: CCACAACATACTCAGCACCTGC	NM 204305.1
*ß-actin*	Fwd: GACTCTGGTGATGGTGTTAC Rev: AGCACAGCTTCTCCTTGATG	NM 205518.1
*CRK*	Fwd: CGGGTTATCCAGAAGCGAGTC Rev: TTCTCCTTCCCACTGACCACTC	
**Primers Used in Bisulfite Genomic Sequencing Assay**		
**Primers Name**	**Sequences (5′–3′)**	**GenBank Accession No.**
gga-miR-126 CpGi-1	Fwd-1: GTTGTTTGGTTAGTATAGAGAGAAATTTA Rev-1: ATTTCCTCCCCCCCAARCCA Fwd-2: TTYGGTTATTGTATYGGTGATGGGATTTTA Rev-2: ATCCCTCCRCCCCRACCCAATAAA	NR 031468.1
gga-miR-126 CpGi-2	Fwd-1: GTATAAGTTTAGGTTTTGTAGGG Rev-1: TTCRTACCTTTACTATCAACAAATA Fwd-2: TTGTAGGGGTGATAAAGTTTGGTTG Rev-2: TATCAACAAATAAATTAACACTCATATCTTCC	NR 031468.1
M13	Fwd: GTAAAACGACGGCCATG Rev: CAGGAAACAGCTATGAC	
**Primers Used in *EGFL-7* 3′ RACE Analysis**		
**Primers Name**	**Sequences (5′–3′)**	**Position on** **Chromosome 17**
ex8-1	Fwd: ATCATCTCCAGGAAGGCACAAGGTA	8230922-8230898
ex8-2	Fwd: AAAGAGAAGGCTGGTTTCCCTCATC	8230896-8230872
ex8-3	Fwd: GTTTACACTGGCACAGCACCAGCT	8230443-8230420
ex8-4	Fwd: CTCCCCAGCTCTGTTTGCTCAAG	8230362-8230340
**Primers Used in *EGFL-7* Transcript Analysis**		
**Primers Name**	**Sequences (5′–3′)**	**Position on** **Chromosome 17**
ex(-5)-1	Fwd: CCACGAGGCAGTGTGGCAGT	8245224-8245205
ex(-5)-2	Fwd: ACGAGGCAGTGTGGCAGTGG	8245222-8245203
ex(-4)-1	Fwd: AGCAAGTAGTCCCAGTTCTGAGC	8243008-8242986
ex(-3)-1	Fwd: CAGTGAGAAACCAAGATGCTCCT	8241801-8241779
ex(-2)-1	Fwd: GTGATGGGATTTCACGGTAGC	8240504-8240484
ex(-2)-2	Fwd: CAGGAGAGCTGCTCTGCGAG	8240422-8240403
ex(-1)-1	Fwd: AGGGACCGACTCGGCCTGG	8240112-8240094
ex(-1)-2	Fwd: CGGCTCCGCCACCGCCAC	8240049-8240032
ex1-1	Rev: GGCTCCTACTGCATGGCTTG	8237474-8237493
ex1-1	Fwd: GAACAGCAAGCCATGCAGTAG	8237499-8237479
ex1-2	Fwd: AAGCCATGCAGTAGGAGCCCAC	8237492-8237471
ex 8-1	Rev: CAGCCTTCTCTTTCTACCTTGTG	8230884-8230906
ex8-2	Rev:CCTTGTGCCTTCCTGGAGATGA	8230900-8230921
**Primers for Plasmid Constructs**		
**Primers Name**	**Sequences (5′–3′)**	**GenBank/miRBase Accession No.**
gga-miR-126 *Pme*I	Fwd: GACT*GTTTAAAC*GCACATCCATCCGAGCCACAAG Rev: GACT*GTTTAAAC*GAGCATGTAGATGGCTCTCCCAG	NR 031468.1
gga-miR-21 *Pme*I	Fwd: GACT*GTTTAAAC*TGAATGTCCTCCTGTGTTGCCAG Rev: GACT*GTTTAAAC*CTGGAGATGGGTGAGCAAACG	MI0004994
MDV1-miR-M7 *Pme*I	Fwd: GACT*GTTTAAAC*GATGCTCTCTAGCCAAGAGAG Rev: GACT*GTTTAAAC*GCAGTTCTGAGGACACATTT	MI0005099
gga-miR-155 *Pme*I	Fwd: GACT*GTTTAAAC*CTAGAGTTCTTCTGTAGGCTGTATG Rev: GACT*GTTTAAAC*GAGTTCTGATGAGAGGCATGGTAC	MI0001176
**Primers for tetR**		
**Primers Name**	**Sequences (5′–3′)**	
tetR	Fwd: GATGTTAGATAGGCACCATACTC Rev: GTAGTAGGTGTTTCCCTTTCTTC	
