# Supplementary material for: Epigenetic Silencing of MicroRNA-126 Promotes Cell Growth in Marek’s Disease
Source: Microorganisms. 2021 Jun 21;9(6):1339. doi: 10.3390/microorganisms9061339 (PMC8235390; doi:10.3390/microorganisms9061339)
Supplement: Supplementary file 1 [file microorganisms-09-01339-s001.zip › microorganisms-1232556-supplementary.pdf]

Supplementary Materials

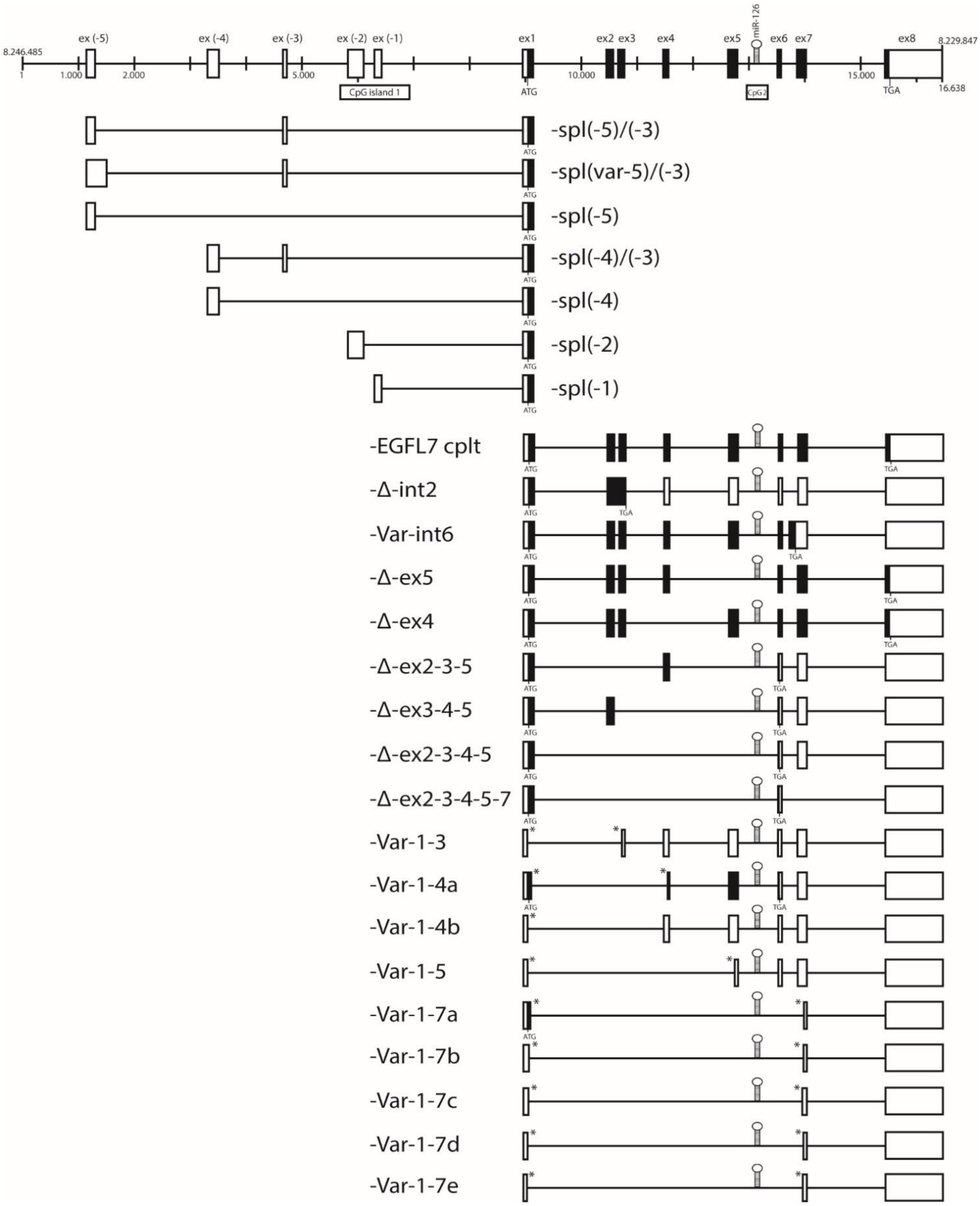

Figure S1. Schematic representation of all transcripts encoding the epidermal growth factor like-7 (*EGFL-7*) gene. The full-length *EGFL-7* gene is represented on top of the image. Gene structure and exon annotations were established from bioinformatics predictions available for *EGFL-7*. Alternative transcripts from exon −5 to exon 1 and alternative transcripts from exon 1 to exon 8 are shown. Non-coding exons are represented by white boxes. Coding exons are represented by black boxes. The black bars are introns. MiR-126 is represented by a stem-loop structure in the fifth intron. * represents unconventional splice sites (alternative donor or acceptor).

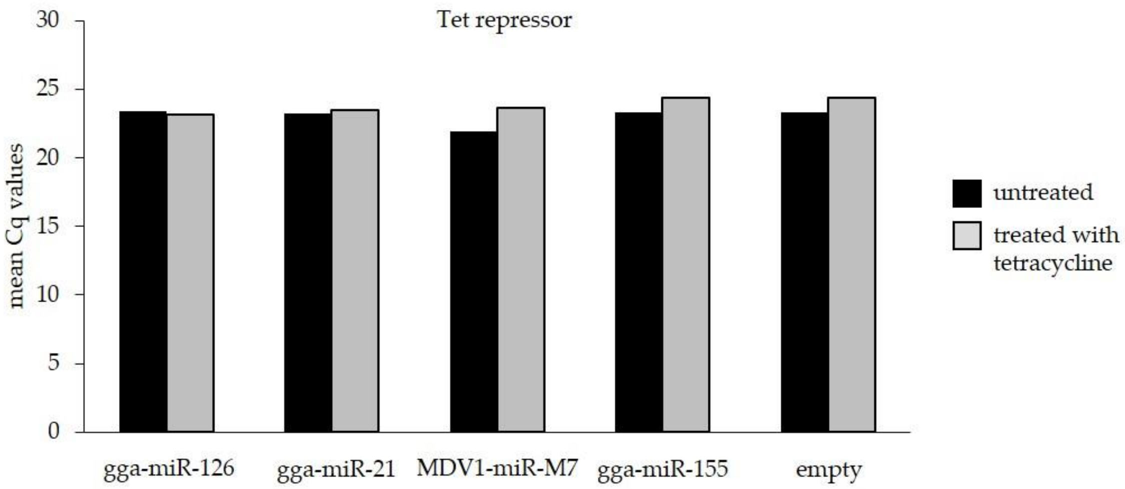

(a)

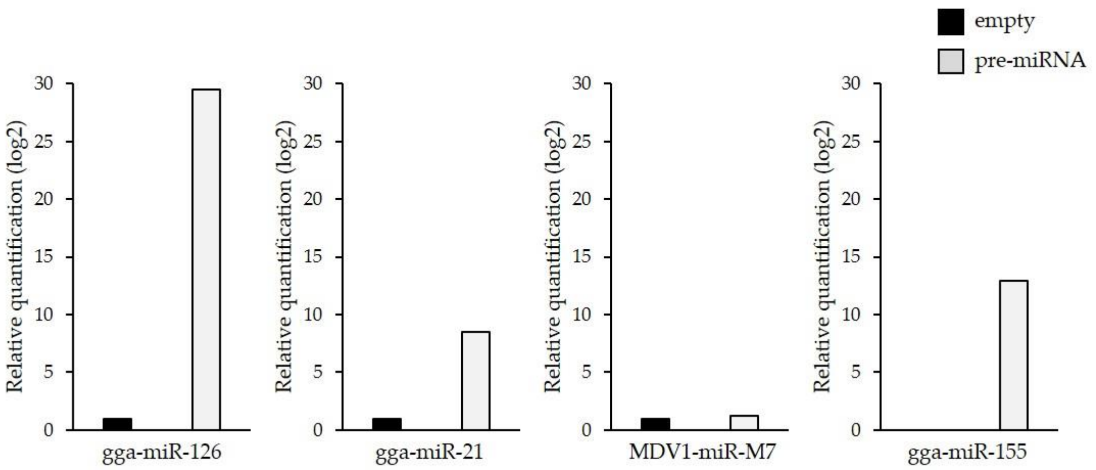

(b)

Figure S2. Quantification of the tet repressor and different micro-RNAs in the stable MSB-1 cell lines treated with tetracycline. (**a**) Quantification by qRT-PCR of the tet repressor (tetR) in treated (light grey) and untreated (black) stable cell lines. The X-axis represents the different cell lines containing the plasmid, allowing the overexpression of miR-126 and control miRNAs, as well as a plasmid without pre-miRNA sequence inside (empty). The y-axis represents the mean of a technical triplicate of raw Cq values. (**b**) Quantification by qRT-PCR of the expression level of the miRNAs within their respective cell lines 48 h after the induction of their expression by tetracycline treatment (light grey) relative to the expression of these miRNAs by the cells containing an empty plasmid without pre-miRNA (black).
